# Implementation of Screening for *Toxoplasma gondii* Infection in Pregnancy

**DOI:** 10.4021/jocmr2010.05.321w

**Published:** 2010-06-15

**Authors:** Massimo De Paschale, Carlo Agrappi, Maria Teresa Manco, Teresa Cerulli, Pierangelo Clerici

**Affiliations:** a Microbiology Unit, Hospital of Legnano, Milan, Italy

## Abstract

**Background:**

Since 1998, Italian law requires serological screening for toxoplasmosis by the thirteenth week of pregnancy, and seronegative women should undergo further checks every 30 - 40 days until delivery (a total of 5 - 7 screenings). This is an improvement of the previous law which foresaw three free tests (one by the end of the third month, one in the fifth, and one in the ninth month of pregnancy). The aim of this study was to assess the implementation, in an urban area of Northern Italy, of the 1998 law about 10 years after its entry into force.

**Methods:**

Of the 4,694 women who initiated and completed a pregnancy in the period 2006 - 2008, we recorded the trimester of pregnancy in which they underwent their first screening, the total and average number of screenings during pregnancy, and the trimester distribution of the screenings.

**Results:**

A total of 84.1% of the women underwent their first screening during the first trimester. The negative cases underwent an average of 3.7 screenings during pregnancy, with 34.9% undergoing five or more. Sixty percent of the women underwent at least one screening per trimester.

**Conclusions:**

Our data indicates active screening during the first trimester, but fewer screenings than required by law during pregnancy as a whole. Therefore further efforts are needed to improve screening implementation.

**Keywords:**

Anti-*Toxoplasma* antibodies; Congenital infection; Pregnancy trimester; *Toxoplasma* screening; Seroconversion; Seronegative women; Seropositive women; Screening protocol

## Introduction

Infection due to *Toxoplasma gondii* is one of the major causes of congenital infection leading to severe fetal damage [1]. It is estimated that congenital toxoplasmosis affects 1 - 10/10,000 babies in Europe [2], but its incidence and severity vary depending on the trimester in which the infection is contracted: the frequency of transmission increases proportionately with gestational age, whereas severity of infection decreases [3-5].

In addition to health education campaigns, preventive screening programmes have been proposed for pre-pregnant and pregnant women, as well as newborns, but depending on the prevalence of infection in the population, cost/benefit questions mean that serological screening during pregnancy is not recommended in some countries (Britain, Holland, Norway and USA) [6-9], and is implemented in different ways in others such as France, Belgium, Switzerland, Germany and Austria [10-12]. In Italy, where the reported antibody prevalence varies from 21% to 48% [13-18], the law of 1995 (Official Gazette No. 87, 13/04/95) foresaw three free tests for *Toxoplasma* antibodies (one by the end of the third month, one in the fifth, and one in the ninth month of pregnancy). Since 1998 (Official Gazette No. 245, 20/10/98), there has laid down a protocol based on an initial screening by the end of the 13th week, and the further screenings of seronegative women every 30 - 40 days until delivery (a total of 5 - 7). Implementing such a demanding screening program requires the full cooperation of general practitioners and women, as well as an efficient organisation capable of providing easy access for sampling and rapid results. Furthermore, it has been shown that 30 - 35% of seronegative women do not complete the follow-up during pregnancy, regardless of whether screening is formally recommended or not [19, 20].

The aim of this study was to assess the implementation of serological *Toxoplasma* antibody screening during pregnancy in an urban area of Northern Italy about 10 years after entry into force of 1998 law.

## Materials and Methods

We retrospectively reviewed the data concerning 4,694 women (mean age 31.4 years; range 15 - 49 years) resident in the urban area of Legnano, near Milan (Northern Italy), who had started and concluded a pregnancy in the period 2006 - 2008, when women underwent serological screening for antibodies to *Toxoplasma* IgG and IgM ELISA (Enzyme-Linked ImmunoSorbent Assay) (ETI-TOXOK-G-PLUS, ETI-TOXOK reverse M-PLUS; Dia Sorin, Saluggia, Italy). The IgG cut-off value was 15 IU/m; in the case of IgM, samples with a D.O. sample/D.O. cut off value of no less than 1 were considered positive.

The ELISA IgM-positive samples were subsequently tested by means of ELFA (Enzyme-Linked Fluorescent Assay) (VIDAS Toxo IgM, BioMerieux, Lyon, France); the samples with an index of no less than 0.65 were considered positive, and those with an index of 0.55 - 0.65 were considered borderline. When possible, IgG avidity was determined in the ELISA IgM-positive samples (VIDAS Toxo IgG avidity, BioMerieux, Lyon, France), and was classified low if the index was less than 0.200, borderline if it was no less than 0.200 but less than 0.300, and high if it was no less than 0.300. The IgM-positive women were sent to Reference Centres.

The considered data were the trimester of pregnancy in which the women underwent their first screening, the total and average number of screenings during pregnancy, and the trimester distribution of the screenings.

## Results

Of the 4,694 women involved in the study, 3,949 underwent their first screening in the first trimester (84.1% = group 1), 431 in the second (9.2% = group 2), and 314 in the third (6.7% = group 3).

At the first screening, a total of 3,634 women (77.4%) were negative for both anti-*Toxoplasma* IgG and IgM antibodies, 974 (20.7%) were IgG positive and IgM negative, and 86 (1.8%) were IgM positive, of whom 14 (0.3%) were IgG negative and 72 (1.5%) IgG positive. Table 1 shows the seroprevalence of antibodies in relation to the trimester of the first screening.

**Table 1:** Prevalence of Anti-Toxoplasma Antibodies by Trimester of First Screening

	I trimester (group 1)		II trimester (group 2)		III trimester (group 3)		Total	
Anti-Toxoplasma antibodies	No.	% (95% CI)	No.	% (95% CI)	No.	% (95% CI)	No.	% (95% CI)
IgG neg/IgM neg	2975	75.3 (73.95-76.65)	386	89.6 (86.72-92.48)	273	86.9 (83.17-90.63)	3634	77.4 (76.20-78.60)
IgG pos/IgM neg	893	22.6 (21.29-23.90)	44	10.2 (7.34-13.06)	37	11.8 (8.23-15.37)	974	20.7 (19.54-21.86)
IgG neg/IgM pos	12	0.3 (0.13-0.47)	0	0 (0.00-0.00)	2	0.6 (0.00-1.45)	14	0.3 (0.14-0.46)
IgG pos/IgM pos	69	1.7 (1.30-2.10)	1	0.2 (0.00-0.62)	2	0.6 (0.00-1.45)	72	1.5 (1.15-1.85)
Total	3949		431		314		4694	

CI: Confidence Interval

Of the 86 ELISA IgM-positive samples, 59 (68.6%) were positive or borderline at ELFA: four IgG- negative and 55 IgG-positive samples, in which IgG avidity was low in six cases (10.9%), borderline in four (7.3%) and high in 45 (81.8%). Fourteen cases were considered primary infections: four IgG negative and IgM positive (confirmed by ELFA), and 10 both IgG and IgM positive (confirmed by ELFA) with low or borderline IgG avidity. The 3,634 seronegative women underwent an average of 3.7 screenings (range 1 - 10); only 1268 (34.9%) underwent five or more as laid down in the Ministerial Decree (Table 2), but 2,181 (60.0%) underwent at least one screening per trimester (Table 3).

**Table 2:** Average and Total Number of Screenings of Pregnant Seronegative Women by the Trimester of the First Screening

Trimester of first screening	No. of seronegative women	Average number of screenings (range)	No. of screenings		
			1	2 - 4	≥ 5
1	2975	4.1 (1 - 10)	313 (10.5%)	1415 (47.6%)	1247 (41.9%)
2	386	2.3 (1 - 6)	118 (30.6%)	247 (64.0%)	21 (5.4%)
3	273	1.2 (1 - 3)	230 (84.2%)	43 (15.8%)	-
Total	3634	3.7 (1 - 10)	661 (18.2%)	1705 (46.9%)	1268 (34.9%)

**Table 3:** Screenings of Seronegative Women by Trimester of Pregnancy

Screenings	No. of women	% (95% CI)
Only I trimester	353	9.7 (8.74 - 10.66)
I trimester + II trimester	300	8.3 (7.40 - 9.20)
I trimester + II trimester + III trimester	2181	60.0 (58.41 - 61.59)
I trimester + III trimester	141	3.9 (3.27 - 4.53)
Only II trimester	138	3.8 (3.18 - 4.42)
II trimester + III trimester	248	6.8 (5.98 - 7.62)
Only III trimester	273	7.5 (6.64 - 8.36)
Total	3634	

CI: Confidence Interval

Of the 2,975 seronegative women who underwent their first screening during the first trimester (group 1), 2,481 (83.4%) underwent at least one screening in the second, and 2,322 (78.1%) in the third. Three seroconversions were observed in the third trimester (two IgG negative and ELISA and ELFA IgM positive, and one ELISA and ELFA IgM positive and IgG positive with low avidity) after respectively 32, 37 and 40 weeks of pregnancy (all were negative in the second trimester) (Table 4). There were no seroconversions in group 2 or 3.

**Table 4:** Cases of Seroconversion by Week of Pregnancy

	Anti-*Toxoplasma* IgM antibodies						
	**Weeks of pregnancy**						
Case	13	18	23	27	32	37	40
1	Neg	Neg	-	Neg	Pos	-	-
2	Neg	Neg	Neg	-	-	Pos	-
3	Neg	Neg	-	-	-	-	Pos

Table 5 shows the 17 primary infections (including seroconversions) by the trimester in which IgM appeared: 11 ELISA/ELFA IgM positive and IgG positive with low or borderline avidity, and six ELISA/ELFA IgM positive and IgG negative.

**Table 5:** Primary Infections by the Trimester in Which IgM Was Detected

	I trimester	II trimester	III trimester	Total
IgM+ IgG+ (low or borderline avidity)	9 (81.8%)	1 (9.1%)	1 (9.1%)*	11
IgM+ IgG-	3 (50%)	0	3 (50%)**	6
Total	12 (70.6%)	1 (5.9%)	4 (23.5%)	17

*seroconversion **two seroconversions

## Discussion

The severity of congenital *Toxoplasma* gondii infection has prompted some countries to implement antibody screening campaigns to detect possible infections at risk of being transmitted to the fetus. Current Italian legislation indicates a protocol for laboratory tests including the search for anti-*Toxoplasma* antibodies at the beginning of pregnancy (if possible before the 13th week) and, in the case of IgG negativity, repeat tests every 30 - 40 days until delivery for a total of 5 - 7 screenings. This protocol is particularly demanding and requires the full cooperation of general practitioners and patients.

This study showed that the vast majority of women resident in our area began monitoring by the end of the first trimester, which is very important because it is easier to assess and manage a possible acute infection in this period and that is when most of our cases of primary infection occurred. However, about 16% of the women delayed the first screening until after the first trimester, and it is these to whom efforts to improve screening should be directed, not least because two of the women who were first screened in the third trimester were IgM positive and showed high IgG avidity. As no previous data were available, it is impossible to distinguish an infection occurring before conception from an acute infection occurring in the first trimester.

About one-third of the seronegative women underwent subsequent screening as laid down in the Ministerial Decree, which suggests that a protocol of 5 - 7 samples may be very demanding as pregnant women face a number of difficulties, including a fear of blood sampling, logistical difficulties, taking time off work, etc. Furthermore, it is possible that their general practitioners may not have been aware of the change in legislation, and may have still been operating on the basis of the previous legislation that required only three screenings: in fact if we consider the seronegative women who underwent screening at least once per trimester, the proportion of women who covered the entire pregnancy was 60%, and almost 80% of those who began the follow-up in the first trimester attended at least one further screenings in both the second and the third trimester.

But if the coverage of pregnancy with only three screenings is very satisfactory, the current protocol whit 5 - 7 screenings at intervals of 30 - 40 days would reveal early seroconversion and allow timely therapeutic measures. From this perspective, the results of this study have to be considered disappointing. The three seroconversions were all detected in the third trimester, and the interval between the last negative test and the first positive test was 35 days in only one case; in the other two, it was respectively 98 and 154 days.

Unfortunately, we have no data regarding the transmission of infection to the fetus because the IgM-positive women were all referred for further investigations to Reference Centres throughout the area.

In conclusion, the results of this study show that the current management of screening is sufficiently active in recruiting women within the first trimester of pregnancy, but weaker in following up seronegative cases as indicated by the Ministerial Decree in force. Further studies are necessary to understand exactly what are the critical points in terms of compliance on which to focus the efforts to improve the screening.
